# The association of women’s experience of abuse in childhood with depression during pregnancy and the role of emotional support as a moderator

**DOI:** 10.1371/journal.pone.0289044

**Published:** 2023-07-26

**Authors:** Yu-Mi Kim, Rora Oh, Sung-Hyun Cho, Kyung Ja June, Ji Yun Lee, Hong-Jun Cho, Young-Ho Khang

**Affiliations:** 1 The Support Team for the Seoul Healthy First Step Project, Seoul, Korea; 2 Department of Preventive Medicine, Hanyang University College of Medicine, Seoul, Korea; 3 School of Public Health, Hanyang University College of Medicine, Seoul, Korea; 4 School of Public Health, Seoul National University, Seoul, Korea; 5 College of Nursing, Research Institute of Nursing Science, Seoul National University, Seoul, Korea; 6 Department of Nursing, Soonchunhyang University, Cheonan, Korea; 7 College of Nursing, Kangwon National University, Chuncheon, Korea; 8 Department of Family Medicine, Asan Medical Center, University of Ulsan College of Medicine, Seoul, Korea; 9 Department of Health Policy and Management, Seoul National University College of Medicine, Seoul, Korea; 10 Institute of Health Policy and Management, Seoul National University Medical Research Center, Seoul, Korea; University of Zurich, SWITZERLAND

## Abstract

**Background:**

This study aimed to examine the prevalence of antenatal depression and experience of abuse during childhood, to analyze the association between having experienced childhood abuse and depression during pregnancy, and to explore the role of emotional support as a moderator of that association.

**Methods:**

In total, 44,770 pregnant women were analyzed from the self-administered registry for risk assessment at community public health centers in Seoul, Republic of Korea, for home visiting service provision between 2015 and 2019. The Edinburgh Postnatal Depression Scale (EPDS) was applied for the assessment of depression. The adjusted effects of childhood abuse experience on antepartum depression according to emotional support as an effect moderator were estimated.

**Results:**

Depression was present in 2,451 pregnant women (5.5%), and 1,506 (3.4%) reported having experienced physical, emotional, or sexual abuse in childhood. After adjustment of covariates, pregnant women who had experienced abuse during childhood had EPDS scores 2.79 points higher than pregnant women without such experiences, and those who lacked emotional support during adulthood had 4.96 points higher than their counterparts. The difference in EPDS scores based on childhood abuse experience among women who reported emotional support (2.86) was larger than the difference in EPDS scores among those with no emotional support (1.91) (P for interaction = 0.0106).

**Conclusions:**

The experience of abuse in early life and emotional support in later life are both independently important for understanding antenatal depression in Korean women. More comprehensive emotional support is needed for pregnant women who experienced abuse in childhood.

## Introduction

Major depressive disorder has an estimated prevalence of 3.2% to 9.2% among women and is 1.5 to 2 times more frequent in women than in men [1, 2]. Pregnancy is a period during which women are particularly vulnerable to experiencing depressive symptom and disorders [3, 4]. Antenatal depression could lead to postnatal depression [5] and perinatal depression may harm maternal and child well-being [6–8].

Child maltreatment may leave long-term negative after-effects on the human mind and body in adulthood. Numerous harmful consequences from childhood abuse have been reported, ranging from physical illness to behavioral and psychological problems [9, 10]. One of the most common and serious consequences of child maltreatment is adolescent and adulthood depression [11, 12].

While definitive conclusions about gender differences in the harmful effects of child maltreatment on depression may not yet be possible [13], girls are more likely to experience child maltreatment than boys [14], and the long-term negative impact of child maltreatment has been considered more detrimental to girls than to boys [15, 16]. Recent systematic review on gender difference between childhood maltreatment and depression have not reached firm conclusions, and all primary literature used were the results of Western studies [17].

The association of child abuse with adulthood depression among the general population has been well established [11]. While the literature on the harmful effect of child maltreatment on perinatal depression has been accumulated [18, 19], there is a relative paucity of evidence regarding the association of experience of abuse in childhood with antenatal depression in adulthood in East Asian countries such as Republic of Korea with a history of the Confucian ideal of separation of the roles of women and men [20]. It has been suggested that childhood adverse experiences would be very salient during pregnancy as a woman prepares to be a mom and is developing her maternal identity, and could subsequently make pregnant women particularly vulnerable to mental health issues [21]. Thus, it is imperative to investigate the link between childhood maltreatment experience and antenatal depression, especially in East Asian countries.

Social support, especially emotional support, may be an important protective factor for those who have experienced abuse during childhood against the development of adulthood depression [22–24]. A recent study employing marginal structural models provided evidence that sustained social support, particularly emotional support, displayed protective effects on perinatal depression [25]. A narrative systematic review elaborated that the availability of internal coping mechanisms and external resources could contribute to better outcomes for women who have experienced childhood adversity during the perinatal period [26]. We considered that emotional support would be especially more important among ‘pregnant’ women with childhood maltreatment experience than other types of social support (e.g., instrumental support). It is worth investigating whether emotional support would act similarly as the protective factor against perinatal (especially antenatal) depression among East Asian pregnant women with a different history of gender role from western countries.

With a relatively large dataset of administrative records from 44,770 pregnant women in Seoul, Republic of Korea, this study aimed to examine the prevalence of antenatal depression and experience of abuse in childhood, to analyze the association between having experienced childhood abuse and depression during pregnancy, and to explore the role of emotional support as a moderator of this association. We considered that emotional support rather than other types of social support (e.g., instrumental support) would be particularly important as a moderator in the association of childhood abuse experience with adulthood depression among women in pregnancy. We hypothesized that experiencing abuse in childhood would be closely associated with antenatal depression and that the magnitude of the association would be lower among those with emotional support.

## Methods

### Study subjects

In an attempt to support families during pregnancy or with newborn babies, to improve the health and development of babies, and eventually to close the gap in child development, the Seoul Healthy First Step Project (SHFSP) was launched in 2013 in Seoul, Republic of Korea [27, 28]. The SHFSP, which is an early childhood intervention program based on nurse home visits, was the first governmental endeavor of this type in the Republic of Korea. In 2019, the SHFSP covered 31.8% of families with newborn babies in Seoul [29].

Between 2013 and 2019, 86,561 families in Seoul have been registered using a self-administered baseline registration form containing information for the primary risk assessment for service provision at local public health centers. Among them, the study subjects were limited to 44,770 pregnant women who were enrolled between 2015 and 2019, completed the Edinburgh Postnatal Depression Scale (EPDS), and provided information on all the study variables described below.

Each study subject provided written informed consent. The institutional review board of Kangwon University approved the study, which analyzed secondary data excluding personal identifiers (KWNUIRB-2020-06-003). This study was conducted in accordance with the World Medical Association Declaration of Helsinki.

### Measurement of depression during pregnancy

Depressive affect was measured with the 10-item Edinburgh Postnatal Depression Scale (EPDS), which is recommended for depression screening in peripartum women [30]. A validated Korean version was employed [31]. The EPDS consists of 10 individual items, the score for each of which ranges from 0 to 3 points; therefore, the total score ranges from 0 to 30. A cut-off value of 13 or higher was reported to yield sensitivity and specificity of 0.66 (95% confidence interval [CI], 0.58–0.74) and 0.95 (95% CI, 0.92 to 0.96) [32]. The Cronbach α coefficient, measuring internal consistency among question items, was calculated as 0.84 in this study population.

### Measurement of experience of abuse in childhood and emotional support

Variables for childhood abuse experience and emotional support were based on the psychosocial assessment in SHFSP (see S1 Table). The psychosocial questions in SHFSP were originally taken from SAFE START guideline in NSW, Australia [33]. Information on experience of childhood abuse was obtained through the question “Have you ever been physically, emotionally, or sexually abused in your childhood?” with responses of “yes” or “no.” The question “Do you have someone you are able to talk to about your feelings or worries?” was used as an indicator for emotional support, with “yes” or “no” responses. Although a variable for instrumental support existed in our data, we focused on emotional support because we considered that the emotional support is particularly important as a moderator in the association between childhood abuse experience and depression during pregnancy. S2–S4 Tables provided the additional analysis results when the instrumental support was employed.

### Other covariates

The following covariates were obtained from self-administered baseline registration forms provided by the pregnant women: maternal age, gestational age (weeks), whether participants were national basic living security program recipients (a measure for poverty), physical and mental disability, whether participants were single parents, whether participants were marriage migrant women, current smoking, alcohol drinking, and past treatment history for emotional issues. A variable for parity (e.g., primiparity) was unavailable in the data obtained prenatally.

### Statistical analysis

Sociodemographic characteristics, maternal health behaviors, and the past medical history of study subjects were presented according to whether they had experienced abuse during childhood. The descriptive statistics for antepartum depression and emotional support were also compared according to childhood abuse experience. In the univariable analysis, the association between antepartum depression and each variable related to study subjects’ characteristics was assessed using a general linear model, and coefficients were estimated accordingly. For maternal age, which is a continuous variable, the most appropriate function for its relationship with antepartum depression was determined based on the results in a generalized additive model. In the multivariable analysis, all variables considered in the univariable analysis were adjusted, and the existence of significant interaction effects was tested at an alpha level of 0.05. As the interaction effect was statistically significant, the adjusted effects of childhood abuse experience on antepartum depression according to emotional support as a moderator were estimated using least square means as follows.–Assuming the following multivariable model:

Edinburgh Postnatal Depression Scale scores = b0 + b1*presence of Emotional support + b2* presence of Childhood abuse experience + b3* presence of Emotional support* presence of Childhood abuse + c*other covariates + errors.

The adjusted effects of childhood abuse experience in the presence of emotional support are derived as (b0 + b1 + b2 +b3 + c*other covariates)- (b0 + b1+ c*other covariates) = b2+b3. In the same way, the adjusted effects of childhood abuse experience in the absence of emotional support are calculated as (b0 + b2 + c*other covariates)- (b0 + c*other covariates) = b2.

As a sensitivity analysis (S5 Table), the effects of childhood abuse experience and emotional support on the probability of EPDS 10 points or higher were estimated as follows.

Assuming the following multiple logistic regression model:

logit(probability of EPDS 10 points or higher) = b0 + b1*presence of Emotional support + b2* presence of Childhood abuse experience + b3* presence of Emotional support* presence of Childhood abuse + c*other covariates = XB.

As the probability of EPDS 10 points or higher are calculated as 1/{1 + exp(-XB)}, the adjusted effects of childhood abuse experience in the presence of emotional support are derived as 1/{1 + exp(-b0—b1—b2 -b3—c*other covariates) - 1/{1 + exp(-b0—b1—c*other covariates). Analogously, the adjusted effects of childhood abuse experience in the absence of emotional support are estimated as 1/{1 + exp(-b0—b2—c*other covariates) - 1/{1 + exp(-b0—c*other covariates).

All statistical analyses were conducted using SAS version 9.4 (SAS Institute, Cary, NC, USA).

## Results

A total of 1,506 subjects (3.4%) among the 44,770 pregnant women answered “yes” to the self-administered question on the experience of physical, emotional and sexual abuse in childhood. The average maternal age among all study subjects was 32.6 ± 4.0 years, and the mean gestational age of the pregnant women was 20.8 ± 11.2 weeks. Women who experienced abuse in childhood were more likely to be national basic living security program recipients, single parents, and marriage migrant women, and also had higher prevalence rates of physical and mental disabilities, current smoking, alcohol drinking, and past treatment history for emotional issues (all P-values < 0.05) (Table 1).

**Table 1:** Characteristics of study subjects according to childhood abuse experience among 44,770 pregnant women in Seoul, Republic of Korea.

		Childhood abuse experience		
	All	Response of "No"	Response of "Yes"	
	N = 44,770	N = 43,264 (96.6%)	N = 1,506 (3.4%)	P-value
**Age (years)**				
Mean ± SD	32.56 ± 3.99	32.58 ±3.94	32.01 ± 5.09	< .0001
Median [min, max]	33 [14, 45]	33 [15, 45]	33 [14, 45]	
**Gestational age (weeks)**				
Mean ± SD	20.82 ±11.24	20.81 ±11.25	21.15 ±10.87	0.4074
Median [min, max]	19 [1, 42]	19 [1, 42]	20 [3, 40]	
**National basic livelihood security program recipient, n (%)**				< .0001
Not applicable	44,380 (99.13%)	42,951 (99.28%)	1,429 (94.89%)	
Recipients	390 (0.87%)	313 (0.72%)	77 (5.11%)	
**Disability, N (%)**				< .0001
Physical disability	90 (0.20%)	81 (0.19%)	9 (0.60%)	
Mental disability	26 (0.06%)	15 (0.03%)	11 (0.73%)	
No	44,654 (99.74%)	43,168 (99.78%)	1,486 (98.67%)	
**Single parents, N (%)**				< .0001
No	44,510 (99.42%)	43,061 (99.53%)	1,449 (96.22%)	
Yes	260 (0.58%)	203 (0.47%)	57 (3.78%)	
**Marriage migrant women, N (%)**				0.0269
No	44,046 (98.38%)	42,575 (98.41%)	1,471 (97.68%)	
Yes	724 (1.62%)	689 (1.59%)	35 (2.32%)	
**Current smoking, N (%)**				0.0006
No	44,652 (99.74%)	43,158 (99.75%)	1,494 (99.20%)	
Yes	118 (0.26%)	106 (0.25%)	12 (0.80%)	
**Alcohol drinking, N (%)**				0.0082
No	44,579 (99.57%)	43,086 (99.59%)	1,493 (99.14%)	
Yes	191 (0.43%)	178 (0.41%)	13 (0.86%)	
**Past treatment history for emotional issues, N (%)**				< .0001
No	43,024 (96.10%)	41,826 (96.68%)	1,198 (79.55%)	
Yes	1,746 (3.90%)	1,438 (3.32%)	308 (20.45%)	

N: number, SD.: standard deviation, Min: minimum, Max: maximum

The absence of emotional support was 5.8 times more common among women who had experienced childhood abuse than among those who had not (9.10% versus 1.57%, P < 0.0001). The mean EPDS score was 9.1 ± 5.5 among those who had experienced childhood abuse, but was 5.1 ± 3.9 among those who had not (P < 0.0001). When an EPDS score cut-off of 13 or higher was used, the prevalence of antenatal depression was 24.3% among pregnant women who had experienced child abuse, whereas it was 4.8% among those who had not (P < 0.0001) (Table 2).

**Table 2:** Distribution of emotional support and depression during pregnancy according to child abuse experience among 44,770 pregnant women in Seoul, Republic of Korea.

		Childhood abuse experience		
	All	Response of "No"	Response of "Yes"	
	N = 44,770	N = 43,264 (96.6%)	N = 1,506 (3.4%)	P-value
**Emotional support, N (%)**				< .0001
No	817 (1.82%)	680 (1.57%)	137 (9.10%)	
Yes	43,953 (98.18%)	42,584 (98.43%)	1,369 (90.90%)	
**EPDS score**				< .0001
Mean ± SD	5.26 ± 4.00	5.12 ± 3.87	9.13 ± 5.48	
Median [min, max]	5 [0, 30]	4 [0, 30]	8 [0, 30]	
**Categorization of depression with EPDS score**				< .0001
< 10, N (%)	38,576 (86.16%)	37,701 (87.14%)	875 (58.10%)	
10–12, N (%)	3,743 (8.36%)	3,478 (8.04%)	265 (17.60%)	
13≤, N (%)	2,451 (5.47%)	2,085 (4.82%)	366 (24.30%)	

N: number, SD.: standard deviation, EPDS: Edinburgh Postnatal Depression Scale

The association of maternal age with depression during pregnancy showed the form of a cubic function, with the highest EPDS scores in participants in their teens and early 20s, the lowest scores in those in their late 20s and early 30s, and increasing scores thereafter (Table 3). The univariable analysis showed that demographic characteristics, socioeconomic factors, and maternal health behaviors were associated with the EPDS score (all P-values < 0.0001). Pregnant women in the third trimester had scores that were on average 0.17 points higher than those in the first or second trimester. Pregnant women who were living in poverty (i.e., national basic livelihood security program recipients), had a mental disability, were single parents, had a past treatment history for emotional issues, had no emotional support, and had experienced abuse during childhood had EPDS scores at least 4 points higher than those of their counterparts (P < 0.0001).

**Table 3:** Differences in Edinburgh Postnatal Depression Scale scores (β) according to sociodemographic factors, maternal health behaviors, emotional support, and childhood abuse experience among 44,770 pregnant women in Seoul, Republic of Korea.

	Univariable analysis			Multivariable analysis		
	Β	SE	P-value	β	SE	P-value
**Age**						
Age	-3.48	0.30	< .0001	-1.59	0.29	< .0001
Age, quadratic	0.09	0.01	< .0001	0.04	0.01	< .0001
Age, cubic	0.00	0.00	< .0001	0.00	0.00	0.0004
**Gestational age**						
First or second trimester						
Third trimester	0.17	0.04	< .0001	0.10	0.04	0.005
**National basic livelihood security program recipient**						
Recipients	4.18	0.20	< .0001	0.91	0.21	< .0001
**Disability**						
Physical disability	3.15	0.42	< .0001	1.93	0.40	< .0001
Mental disability	11.33	0.78	< .0001	5.08	0.75	< .0001
**Single parents**						
Yes	6.15	0.25	< .0001	2.36	0.26	< .0001
**Marriage migrant women**						
Yes	0.77	0.15	< .0001	0.34	0.14	0.0175
**Current smoking**						
Yes	3.33	0.37	< .0001	1.29	0.38	0.0006
**Alcohol drinking**						
Yes	1.88	0.29	< .0001	0.88	0.30	0.0028
**Past treatment history for emotional issues**						
Yes	4.18	0.10	< .0001	3.23	0.09	< .0001
**Emotional support**						
No	6.21	0.14	< .0001	4.96	0.14	< .0001
**Child abuse experience**						
Yes	4.01	0.10	< .0001	2.79	0.10	< .0001
**R square**					0.11	

SE: standard error

The adjusted EPDS scores and their standard errors, adjusting for important covariates including age, disability, and socioeconomic status, are presented in Table 3. The magnitude of differences in EPDS scores (beta coefficients) according to study variables including childhood abuse experience and emotional support after simultaneous adjustment of the study variables was attenuated. For example, women living in poverty (i.e., national basic livelihood security program recipients) had EPDS scores 4.18 points higher than those of their counterparts in the univariable analysis, but only 0.91 points higher scores after adjustment of other variables. After controlling for covariates, including emotional support, pregnant women who experienced abuse in childhood had EPDS scores 2.79 points higher than those of pregnant women who had not experienced abuse. The adjusted EPDS score was 4.96 points greater for pregnant women without emotional support when controlling for covariates, including childhood abuse experience (Table 3).

Table 4 shows the results of an analysis of the moderating effect of emotional support in the association of childhood abuse experience with depression during pregnancy. After full adjustment for the covariates considered in this study, the lowest adjusted depression score (EPDS) was found among pregnant women with emotional support but without childhood abuse experience (4.94 points), while the highest score was found among those without emotional support but with childhood abuse experience (11.96 points), corresponding to a difference of 6 points (model 3 in Table 4). A 2.86-point difference in the EPDS score was found between groups with and without childhood abuse experience among pregnant women with emotional support, while a 1.91-point difference was detected among those without emotional support. The interaction between emotional support and child abuse experience was statistically significant at the 0.05 level in model 3 with full statistical adjustment (P = 0.0106).

**Table 4:** Adjusted Edinburgh Postnatal Depression Scale scores according to emotional support and childhood abuse experience among 44,770 pregnant women in Seoul, Republic of Korea.

Emotional support	Childhood abuse experience	Model 1	Model 2	Model 3
		LSMEAN [95% CI]	LSMEAN [95% CI]	LSMEAN [95% CI]
Yes	No	5.03 [4.99, 5.07]	4.87 [4.85, 4.90]	4.94 [4.92, 4.97]
Yes	Yes	8.62 [8.42, 8.82]	8.31 [8.10, 8.51]	7.80 [7.60, 8.00]
Difference		3.59 [3.38, 3.80]	3.43 [3.23, 3.64]	2.86 [2.65, 3.07]
P-value for difference		< .0001	< .0001	< .0001
No	No	10.77 [10.48, 11.06]	10.48 [10.19, 10.77]	10.05 [9.76, 10.33]
No	Yes	14.23 [13.58, 14.87]	13.74 [13.10, 14.39]	11.96 [11.32, 12.60]
Difference		3.46 [2.75, 4.16]	3.26 [2.56, 3.97]	1.91 [1.21, 2.61]
P-value for difference		< .0001	< .0001	< .0001
P-values for the interaction between childhood abuse experience and emotional support		0.7246	0.6519	0.0106

LSMEAN: least square mean in the Edinburgh Postnatal Depression Scale score, CI: confidence interval

Model 1: univariable analysis without any adjustment; model 2: adjusted for age; model 3: adjusted for age, gestational age, national basic living security program recipient status, disability, single parenting, marriage migrant women, current smoking, alcohol drinking, and past treatment history for emotional issues.

## Discussion

Of 44,770 pregnant women aged 14–45 living in Seoul, Korea, 2,451 (5.5%) had depression when using an EPDS cut-off value of 13 or higher. The prevalence of depression during pregnancy in this study is slightly lower than previous estimates [3, 4]. The prevalence of antenatal depression was lower than the prevalence of postpartum depression [28]. Of those with antenatal depression (EPDS score being 13 or higher), only 18.1% had past treatment history for emotional issues (data not shown here). In total, 1,506 (3.4%) of the study subjects reported having experienced physical, emotional, or sexual abuse in childhood. In high-income countries, the prevalence of physical abuse is estimated to be as high as 4–16% per year, and sexual abuse in childhood is estimated to affect 30% of girls and 15% of boys [14]. The self-reported prevalence in this study might have been underestimated. A cultural tolerance for physical abuse as a form of educational discipline [34] and a tendency to conceal sexual abuse [35] might have contributed to the relatively low estimate of lifetime prevalence of childhood abuse experience in this study. Since the data were collected for administrative purposes in the SHFSP, many pregnant women who had experienced abuse during childhood might have been reluctant to disclose their experiences because their reports could affect the service delivery of the SHFSP.

The results of this study showed associations of experience of abuse in childhood and emotional support with antenatal depression. The average depression score measured by the EPDS among pregnant women who had experienced abuse during childhood was about 4 points higher than that of those who had not experienced abuse during childhood, which resulted in a 5-fold difference in the prevalence of antenatal depression (4.82% in women without childhood abuse experience versus 24.3% in women with such experience) (Table 2). After adjusting for covariates (age, gestational age, maternal health behavior and mental health issues, and emotional support), the effect of childhood abuse experience on depression during pregnancy was still evident (β = 2.79, P < 0.001) (Table 3). This was the novel result that revealed that the childhood abuse experience is a risk factor for depression of pregnant women in East Asian countries.

In addition, 9.1% of pregnant women who had experienced abuse during childhood reported not having emotional support, whereas only 1.6% of those without childhood abuse experience had no emotional support. This result indicates that disadvantages accumulated over the life course among those who had experienced abuse during childhood, who also had higher prevalence rates of antenatal depression and a lack of emotional support. Moreover, having experienced abuse during childhood was also positively associated with receiving support from the national basic living security program, disabilities, current smoking and alcohol drinking, and a past treatment history for emotional issues. These results are not surprising given the reports of many previous studies on the negative effects of abuse during childhood over the life course and the clustering patterns of disadvantages [10].

Social and emotional support for those with adverse childhood experiences has been suggested to be a protective factor against adulthood depression [22, 23] and perinatal depression [25]. Additionally, emotional support showed positive effects on depressive women who had experienced sexual abuse during childhood [24]. The results of this study showed that the experience of abuse in childhood was closely associated with antenatal depression and emotional support was protective against depression among both pregnant women with and without childhood abuse experience. Instrumental support was also important in the association with childhood abuse experience and with depression. However, the prevalence ratio between those without childhood abuse experience and those with childhood abuse experience was much greater in emotional support (1.57% vs 9.10%, prevalence ratio = 5.8) than in instrumental support (8.22% vs 21.58%, prevalence ratio = 2.6) (S2 Table). The magnitude of effect in predicting Edinburgh Postnatal Depression Scale scores was greater in emotional support than in instrumental support. The difference in Edinburgh Postnatal Depression Scale scores by emotional support was 4.96 while the difference by instrumental support was 1.78 in multivariable analysis (S3 Table).

Analysis results also presented that the difference in EPDS scores according to childhood abuse experience tended to be greater among women with emotional support than among with no emotional support (a 2.86-point difference versus a 1.91-point difference) (Table 4), which is contrary to our initial hypothesis. A similar result was found for instrumental support (S4 Table). In other words, emotional support in the environment of pregnant women with childhood abuse experience was less effective in reducing depression scores than emotional support among those without childhood abuse experience. One possible interpretation for this finding is that emotional support might be insufficient and/or have a weaker effect on offsetting depression in pregnant women who had experienced abuse during childhood than among pregnant women without childhood abuse experience. In some cases, women who have experienced childhood abuse may have to depend on their families of origin for support, even though those same families may have been responsible for the abuse. This situation would decrease the effectiveness of that support in mitigating psychopathology. The second explanation might be that EPDS may not have captured other psychopathological distress such as post-traumatic syndrome caused by the trauma. However, other types of psychopathology were not measured in this study. The third explanation might be that other types of disadvantages between childhood and pregnancy (e.g., abuse experience or poor social relations in adolescence) could have increased antenatal depression but decreased the difference in depression scores according to whether participants had experienced abuse during childhood. Considering that the protective effect of social and emotional support on depression found in this study, a more intensive social and emotional support should be provided for pregnant women with childhood abuse experience.

The results of our analysis indicate that experiences of abuse in early life and emotional support in later life are both independently important for understanding antenatal depression in Korean women. These results may suggest that emotional support interventions could alleviate antenatal depression regardless of experiences of abuse during childhood. Intensive interpersonal psychosocial interventions such as nurse-led prenatal and early childhood intensive home visitation programs seemed to have a protective effect on the development of antenatal depression into postpartum depression and on the reduction of perinatal depression [36, 37]. Considering accumulated disadvantages in life, poor social relationships, the relatively frequent absence of emotional support (5.8 times difference in this study), and associated mental and physical suffering among pregnant women who experienced abuse during childhood, it would be very important to provide sufficient social and emotional support for those with experiences of maltreatment in childhood.

This study has several limitations. First, underreporting and uncertainty of recall could be possible, especially regarding experiences of abuse during childhood [14]. Despite the cultural tolerance for physical abuse as a form of educational discipline [34] and a tendency to conceal sexual abuse, the reported prevalence of childhood abuse experience (3.4%) in this study seems to be fairly low. The nature of data collection of this study (i.e., data were obtained for service delivery through the SHFSP) should be considered as an explanation of the low prevalence. Second, emotional support was measured using a simple question asking whether there was someone participants could talk to about their feelings or concerns. The fact that the questionnaire was used for administrative purposes did not allow the use of more detailed social and emotional support measurement tools. Third, this study is based on cross-sectional data and no causal analysis was conducted. The possibility of reverse causality (e.g., depression among pregnant women may reduce emotional support and better recall childhood abuse experience) should be also considered. Despite these shortcomings, these findings regarding the prevalence of abuse during childhood and antenatal depression among a large sample of pregnant women provide indispensable data on epidemiological indicators in the Republic of Korea as an example of a high-income Eastern Asian country. Despite the cross-sectional research design, this study has meaningful implications regarding the negative impact of abuse during childhood on antenatal depression and raises the possibility that antenatal depression might be alleviated through interventions targeting emotional support in adulthood.

## Conclusion

The results of this study suggest that childhood abuse experience might be linked to an accumulation of disadvantages from negative childhood events to adulthood social deprivation. However, the results also indicate room for an intervention that may break the chain between abuse in childhood and depression in pregnancy. Especially in Korean society, which is showing a sharp decrease in the birth rate, it is necessary to implement practical policies and a workable program centered on women and children to mitigate adverse social and emotional experiences associated with pregnancy. Prenatal and early childhood home visiting intervention such as SHFSP may alleviate the maternal depression by improving the maternal self-confidence and self-efficacy.

## Supporting information

S1 Fig LSMEANs of EPDS score by emotional support and childhood abuse experience. LSMEAN of EPDS: least square mean of the Edinburgh Postnatal Depression Scale.
(TIF)

S1 Table The eight psychosocial variables and associated questions included in the psychosocial assessment of the Seoul Healthy First Step Project. (DOCX)

S2 Table Distribution of instrumental support and depression during pregnancy according to child abuse experience among 44,770 pregnant women in Seoul, Republic of Korea. (DOCX)

S3 Table Differences in Edinburgh Postnatal Depression Scale scores (β) according by instrumental support among 44,770 pregnant women in Seoul, Republic of Korea. SE: standard error.
(DOCX)

S4 Table Adjusted Edinburgh Postnatal Depression Scale scores according to instrumental support and childhood abuse experience among 44,770 pregnant women in Seoul, Republic of Korea. LSMEAN: least square mean in the Edinburgh Postnatal Depression Scale score, CI: confidence interval. Model 1: univariable analysis without any adjustment; model 2: adjusted for age; model 3: adjusted for age, gestational age, national basic living security program recipient status, disability, single parenting, marriage migrant women, current smoking, alcohol drinking, and past treatment history for emotional issues.
(DOCX)

S5 Table The probability of possible depression (Edinburgh Postnatal Depression Scale scores being 10 points or higher) by emotional support and childhood abuse experience among 44,770 pregnant women in Seoul, Republic of Korea. The probability of EPDS 10 points or higher are calculated as 1/{1 + exp(-XB)} from logit(probability of EPDS 10 points or higher) by multiple logistic regression. CI: confidence interval. Model 1: univariable analysis without any adjustment; model 2: adjusted for age; model 3: adjusted for age, gestational age, national basic living security program recipient status, disability, single parenting, marriage migrant women, current smoking, alcohol drinking, and past treatment history for emotional issues.
(DOCX)
