# Supplementary figures and images for: The association of women’s experience of abuse in childhood with depression during pregnancy and the role of emotional support as a moderator
Source: PLoS One. 2023 Jul 26;18(7):e0289044. doi: 10.1371/journal.pone.0289044 (PMC10370752; doi:10.1371/journal.pone.0289044)

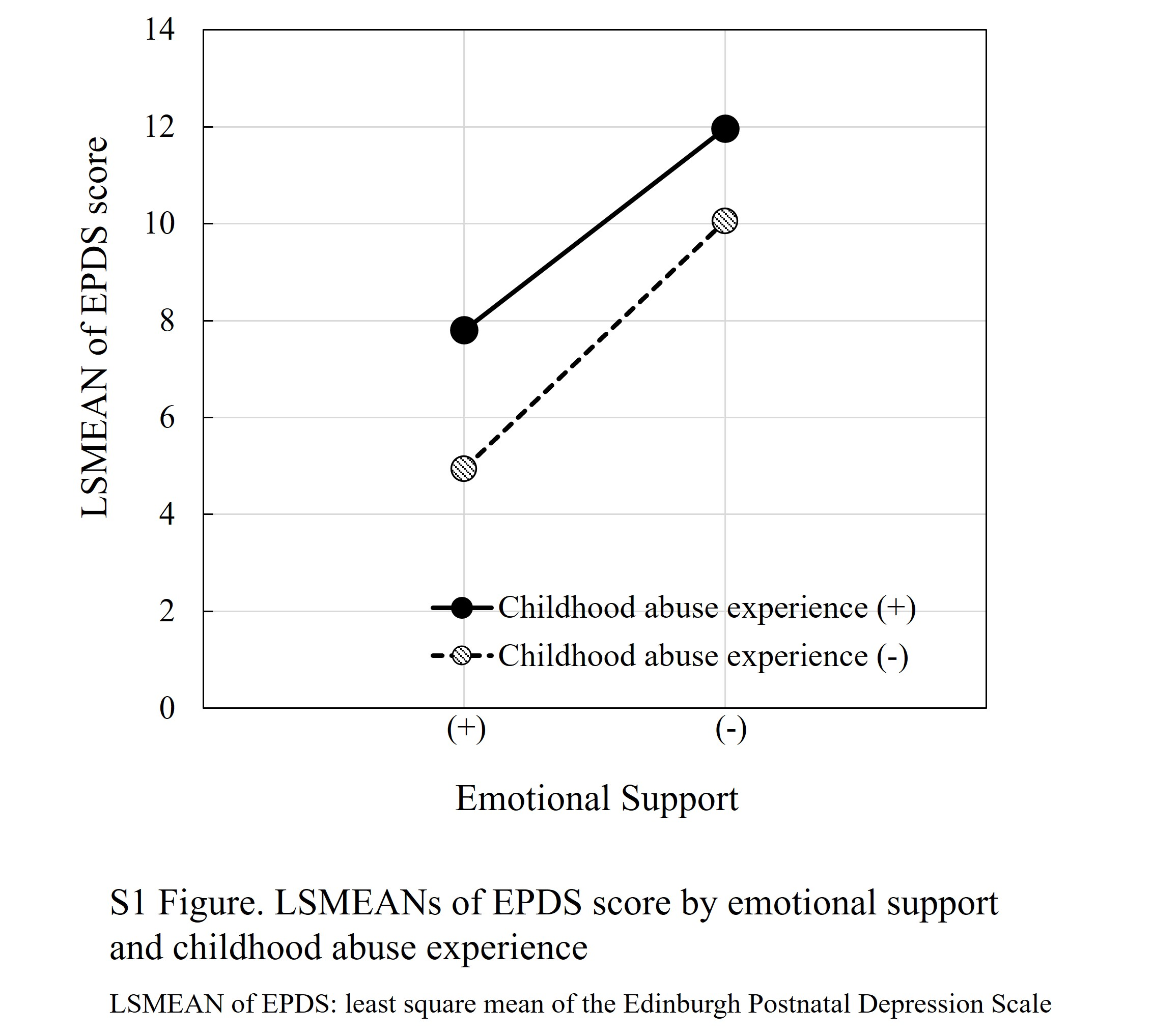

Supplement: S1 Fig — LSMEAN of EPDS: least square mean of the Edinburgh Postnatal Depression Scale. (TIF) [file pone.0289044.s001.tif]
