# Supplementary material for: The association of women’s experience of abuse in childhood with depression during pregnancy and the role of emotional support as a moderator
Source: PLoS One. 2023 Jul 26;18(7):e0289044. doi: 10.1371/journal.pone.0289044 (PMC10370752; doi:10.1371/journal.pone.0289044)
Supplement: S1 Table — (DOCX) [file pone.0289044.s002.docx]

S1 Table. The eight psychosocial variables and associated questions included in the psychosocial assessment of the Seoul Healthy First Step Project

| **Variables** | **Questions in the Seoul Healthy First Step Project** |
| --- | --- |
| The instrumental support | Will you be able to get practical support with your baby? |
| The emotional support | Do you have someone you are able to talk to about your feelings or worries? |
| Recent major stressors in the last 12 months | Have you ever experienced serious stress, change, or loss over the past 12 months such as financial problems, someone close to you dying, or any other serious worries? |
| Low self-esteem | Do you consider yourself a confident person? |
| Past treatment history for emotional issues | Have you ever been treated for emotional issues? |
| Relationship with partner | How would you describe your relationship with your partner? |
| Childhood abuse experience | Have you ever been physically, emotionally, or sexually abused in your childhood? |
| Domestic violence | Do you need any help for domestic violence? |
