# Supplementary material for: The association of women’s experience of abuse in childhood with depression during pregnancy and the role of emotional support as a moderator
Source: PLoS One. 2023 Jul 26;18(7):e0289044. doi: 10.1371/journal.pone.0289044 (PMC10370752; doi:10.1371/journal.pone.0289044)
Supplement: S2 Table — (DOCX) [file pone.0289044.s003.docx]

S2 Table. Distribution of instrumental support and depression during pregnancy according to child abuse experience among 44,770 pregnant women in Seoul, Republic of Korea

|  |  | **Childhood abuse experience** | |  |
| --- | --- | --- | --- | --- |
|  | **All** | **Response of "No"** | **Response of "Yes"** |  |
|  | **N=44,770** | **N=43,264 (96.6%)** | **N=1,506 (3.4%)** | **P-value** |
| **Instrumental support, N (%)** |  |  |  |  |
| No | 3,883 (8.67%) | 3,558 (8.22%) | 325 (21.58%) | <.0001 |
| Yes | 40,887 (91.33%) | 39,706 (91.78%) | 1,181 (78.42%) |  |
| **EPDS score** |  |  |  |  |
| Mean ± SD | 5.26 ± 4.00 | 5.12 ± 3.87 | 9.13 ± 5.48 | <.0001 |
| Median [min, max] | 5 [0, 30] | 4 [0, 30] | 8 [0, 30] |  |
| **Categorization of depression with EPDS score** | | | | <.0001 |
| < 10, N (%) | 38,576 (86.16%) | 37,701 (87.14%) | 875 (58.10%) |  |
| 10–12, N (%) | 3,743 (8.36%) | 3,478 (8.04%) | 265 (17.60%) |  |
| 13≤, N (%) | 2,451 (5.47%) | 2,085 (4.82%) | 366 (24.30%) |  |
