# Supplementary material for: The association of women’s experience of abuse in childhood with depression during pregnancy and the role of emotional support as a moderator
Source: PLoS One. 2023 Jul 26;18(7):e0289044. doi: 10.1371/journal.pone.0289044 (PMC10370752; doi:10.1371/journal.pone.0289044)
Supplement: S3 Table — SE: standard error. (DOCX) [file pone.0289044.s004.docx]

S3 Table. Differences in Edinburgh Postnatal Depression Scale scores (β) according by instrumental support among 44,770 pregnant women in Seoul, Republic of Korea

|  | **Univariable analysis** | | | **Multivariable analysis** | | |
| --- | --- | --- | --- | --- | --- | --- |
|  | **Β** | **SE** | **P-value** | **β** | **SE** | **P-value** |
| **Age** |  |  |  |  |  |  |
| Age | -3.48 | 0.3 | <.0001 | -1.53 | 0.29 | <.0001 |
| Age, quadratic | 0.09 | 0.01 | <.0001 | 0.04 | 0.01 | <.0001 |
| Age, cubic | 0 | 0 | <.0001 | 0.00 | 0.00 | 0.0012 |
| **Gestational age** |  |  |  |  |  |  |
| First or second trimester |  |  |  |  |  |  |
| Third trimester | 0.17 | 0.04 | <.0001 | 0.10 | 0.04 | 0.0068 |
| **National basic livelihood security program recipient** | | | |  |  |  |
| Recipients | 4.18 | 0.2 | <.0001 | 1.15 | 0.21 | <.0001 |
| **Disability** |  |  |  |  |  |  |
| Physical disability | 3.15 | 0.42 | <.0001 | 2.07 | 0.40 | <.0001 |
| Mental disability | 11.33 | 0.78 | <.0001 | 5.65 | 0.76 | <.0001 |
| **Single parents** |  |  |  |  |  |  |
| Yes | 6.15 | 0.25 | <.0001 | 2.66 | 0.26 | <.0001 |
| **Marriage migrant women** |  |  |  |  |  |  |
| Yes | 0.77 | 0.15 | <.0001 | 0.46 | 0.14 | 0.0012 |
| **Current smoking** |  |  |  |  |  |  |
| Yes | 3.33 | 0.37 | <.0001 | 1.30 | 0.38 | 0.0006 |
| **Alcohol drinking** |  |  |  |  |  |  |
| Yes | 1.88 | 0.29 | <.0001 | 0.89 | 0.30 | 0.0026 |
| **Past treatment history for emotional issues** | | | |  |  |  |
| Yes | 4.18 | 0.1 | <.0001 | 3.36 | 0.09 | <.0001 |
| **Instrumental support** |  |  |  |  |  |  |
| No | 2.21 | 0.07 | <.0001 | 1.78 | 0.06 | <.0001 |
| **Child abuse experience** |  |  |  |  |  |  |
| Yes | 4.01 | 0.1 | <.0001 | 2.87 | 0.10 | <.0001 |
| **R square** |  |  |  | 0.10 |  |  |

SE: standard error
