# Supplementary material for: The association of women’s experience of abuse in childhood with depression during pregnancy and the role of emotional support as a moderator
Source: PLoS One. 2023 Jul 26;18(7):e0289044. doi: 10.1371/journal.pone.0289044 (PMC10370752; doi:10.1371/journal.pone.0289044)
Supplement: S4 Table — LSMEAN: least square mean in the Edinburgh Postnatal Depression Scale score, CI: confidence interval. Model 1: univariable analysis without any adjustment; model 2: adjusted for age; model 3: adjusted for age, gestational age, national basic living security program recipient status, disability, single parenting, marriage migrant women, current smoking, alcohol drinking, and past treatment history for emotional issues. (DOCX) [file pone.0289044.s005.docx]

S4 Table. Adjusted Edinburgh Postnatal Depression Scale scores according to instrumental support and childhood abuse experience among 44,770 pregnant women in Seoul, Republic of Korea

| **Instrumental support** | **Childhood abuse experience** | **Model 1** | **Model 2** | **Model 3** |
| --- | --- | --- | --- | --- |
|  |  | LSMEAN [95% CI] | LSMEAN [95% CI] | LSMEAN [95% CI] |
| Yes | No | 4.96 [4.92, 5.00] | 4.79 [4.76, 4.82] | 4.87 [4.85, 4.90] |
| Yes | Yes | 8.57 [8.35, 8.79] | 8.24 [8.02, 8.47] | 7.73 [7.51, 7.95] |
| Difference | | 3.61 [3.39, 3.84] | 3.46 [3.23, 3.68] | 2.86 [2.63, 3.08] |
| P-value for difference | | <.0001 | <.0001 | <.0001 |
| No | No | 6.92 [6.79, 7.04] | 6.72 [6.59, 6.84] | 6.65 [6.52, 6.77] |
| No | Yes | 11.16 [10.74, 11.58] | 10.71 [10.28, 11.13] | 9.57 [9.15, 9.99] |
| Difference | | 4.24 [3.80, 4.68] | 3.99 [3.55, 4.43] | 2.93 [2.49, 3.36] |
| P-value for difference | | <.0001 | <.0001 | <.0001 |
| P-values for the interaction between childhood abuse experience and emotional support | | 0.0128 | 0.0337 | 0.7783 |

LSMEAN: least square mean in the Edinburgh Postnatal Depression Scale score, CI: confidence interval

Model 1: univariable analysis without any adjustment; model 2: adjusted for age; model 3: adjusted for age, gestational age, national basic living security program recipient status, disability, single parenting, marriage migrant women, current smoking, alcohol drinking, and past treatment history for emotional issues.
