# Supplementary material for: The association of women’s experience of abuse in childhood with depression during pregnancy and the role of emotional support as a moderator
Source: PLoS One. 2023 Jul 26;18(7):e0289044. doi: 10.1371/journal.pone.0289044 (PMC10370752; doi:10.1371/journal.pone.0289044)
Supplement: S5 Table — The probability of EPDS 10 points or higher are calculated as 1/{1 + exp(-XB)} from logit(probability of EPDS 10 points or higher) by multiple logistic regression. CI: confidence interval. Model 1: univariable analysis without any adjustment; model 2: adjusted for age; model 3: adjusted for age, gestational age, national basic living security program recipient status, disability, single parenting, marriage migrant women, current smoking, alcohol drinking, and past treatment history for emotional issues. (DOCX) [file pone.0289044.s006.docx]

S5 Table. The probability of possible depression (Edinburgh Postnatal Depression Scale scores being 10 points or higher) by emotional support and childhood abuse experience among 44,770 pregnant women in Seoul, Republic of Korea

| **Emotional support** | **Childhood abuse experience** | **Model 1** | **Model 2** | **Model 3** |
| --- | --- | --- | --- | --- |
|  |  | probability [95% CI] | probability [95% CI] | probability [95% CI] |
| Yes | No | 0.12 [0.12, 0.12] | 0.11 [0.11, 0.12] | 0.11 [0.11, 0.12] |
| Yes | Yes | 0.38 [0.36, 0.41] | 0.35 [0.32, 0.37] | 0.30 [0.27, 0.32] |
| Difference | | 0.26 [0.24, 0.29] | 0.24 [0.22, 0.27] | 0.19 [0.16, 0.21] |
| P-value for difference | | <.0001 | <.0001 | <.0001 |
| No | No | 0.55 [0.52, 0.59] | 0.52 [0.48, 0.56] | 0.49 [0.45, 0.53] |
| No | Yes | 0.77 [0.70, 0.84] | 0.74 [0.65, 0.81] | 0.63 [0.53, 0.72] |
| Difference | | 0.22 [0.14, 0.30] | 0.21 [0.13, 0.28] | 0.14 [0.08, 0.21] |
| P-value for difference | | <.0001 | <.0001 | <.0001 |
| P-values for the interaction between childhood abuse experience and emotional support | | 0.03 | 0.0281 | 0.0063 |

The probability of EPDS 10 points or higher are calculated as 1/{1 + exp(-XB)} from logit(probability of EPDS 10 points or higher) by multiple logistic regression. CI: confidence interval

Model 1: univariable analysis without any adjustment; model 2: adjusted for age; model 3: adjusted for age, gestational age, national basic living security program recipient status, disability, single parenting, marriage migrant women, current smoking, alcohol drinking, and past treatment history for emotional issues
